# The Ameliorative Effect of JNK Inhibitor D-JNKI-1 on Neomycin-Induced Apoptosis in HEI-OC1 Cells

**DOI:** 10.3389/fnmol.2022.824762

**Published:** 2022-03-11

**Authors:** Junling Zhao, Hao Liu, Zhiwei Huang, Ruiming Yang, Liang Gong

**Affiliations:** Department of Otolaryngology, The First Affiliated Hospital of Jinzhou Medical University, Jinzhou, China

**Keywords:** D-JNKI-1, HEI-OC1, neomycin, apoptosis, JNK

## Abstract

Aminoglycosides can cause ototoxicity and lead to hair cell damage. Neomycin-induced ototoxicity is related to increased production of reactive oxygen species (ROS) and triggering hair cell apoptosis. The c-Jun-N-terminal kinase (JNK) pathway plays an essential role during hair cell damage. This study was designed to investigate an inhibitor of JNK, D-JNKI-1 (AM-111/brimapitide) in neomycin-induced HEI-OC1 cell apoptosis. The results demonstrate that neomycin increased intracellular ROS accumulation, which induces apoptosis. D-JNKI-1 decreased neomycin-induced ROS generation, reduced caspase-8 and cleavage of caspase-3 expression, sustained JNK activation and AMPK and p38 phosphorylation, downregulated Bax, and upregulated Bcl-2. Together, D-JNKI-1 plays an essential role in protecting against neomycin-induced HEI-OC1 cell apoptosis by suppressing ROS generation, which inhibited JNK activation and AMPK and p38 phosphorylation to ameliorate JNK-mediated HEI-OC1 cell apoptosis.

## Introduction

Aminoglycosides cause ototoxicity in the inner ear, resulting in hair cell death and causes permanent hearing loss. Neomycin is an aminoglycoside antibiotic administered by mouth for hepatic encephalopathy and surgical prophylaxis (Veirup and Kyriakopoulos, 2022) that is considered the most highly ototoxic. Aminoglycosides induce highly toxic reactive oxygen species (ROS) in the hair cell region and cause hair cell damage (Nadol, 1993).

The mechanism of aminoglycoside-induced hair cell damage involves free radical formation and hair cell oxidative stress (Huang et al., 2000) and also apoptotic cell death pathways. The ROS-initiated hair cell death includes caspase-dependent and caspase-independent apoptosis and necrosis (Chan, 2005; Genestra, 2007). Apoptosis signaling cascades induce mitochondrial ROS release and increase cytosolic ROS. In addition, mitochondrial ROS formation induces the mitochondrial permeability transition pore (PTP) and causes the inner membrane to become permeable to small molecules that lead to apoptosis (Murphy, 2009). Evidence has shown that hair cell apoptosis is the predominant mechanism in aminoglycoside-induced ototoxicity (Huth et al., 2011).

Reactive oxygen species may induce multiple cell death signaling cascades, one being the c-Jun-N-terminal kinase (JNK) pathway, which mediates apoptotic death in response to a variety of stressful stimuli. Evidence has shown that the JNK pathway is involved in neomycin-induced sensory hair cell death (Sugahara et al., 2006; Dhanasekaran and Reddy, 2008). The JNK proteins, in the family of mitogen-activated protein kinases (MAP kinases), are activated by exposure to ototoxic drugs. JNK activation plays an important role in aminoglycoside-induced hair cell death (Matsui et al., 2004). ROS activation of JNK to induce apoptosis has been reported (Davis, 2000); thus, inhibition of the JNK pathway was investigated to reduce aminoglycoside-induced ototoxicity (Pirvola et al., 2000).

D-JNK-1, a cell-penetrating and protease-resistant peptide selectively inhibitor of JNK, was shown to protect hair cells from exposure to aminoglycosides and acoustic trauma (Wang et al., 2003; Kayyali et al., 2018). Therefore, to reduce neomycin-induced hair cell death and better understand the mechanism of D-JNK-1 effects on neomycin-induced hair cell damage, we currently used House Ear Institute-Organ of Corti 1 (HEI-OC1) cells *in vitro* to investigate the role of D-JNK-1 during apoptosis. HEI-OC1 cells, a hair cell-like cell line, are a mouse auditory cell line derived from the auditory organ of a transgenic mouse (Kalinec et al., 2016), which expressed the specific markers for cochlear hair cells and supporting cells, which was used to investigate the aminoglycoside-induced ototoxic mechanism (Kalinec et al., 2003; Chen et al., 2012). We compared neomycin-induced apoptosis at different concentrations, measured the protein expression level of apoptosis-related genes in response to D-JNK-1-treated HEI-OC1 cells after neomycin exposure, and investigated the mechanism of D-JNK-1 inhibited neomycin-induced HEI-OC1 cell apoptosis. Collectively, our findings indicate that D-JNK-1 contributes to protection against neomycin-induced HEI-OC1 cell apoptosis-related to the caspase-mediated apoptotic cascade, the AMPK and JNK pathways.

## Materials and Methods

### Experimental Procedures

House Ear Institute-Organ of Corti 1 (HEI-OC1 cell, RRID:CVCL_D899) was cultured and used in the following experiments. Neomycin was applied to HEI-OC1 cells to induce cell apoptosis. The concentrations of 0, 0.5, 1.0, 2.0, and 5.0 mM neomycin (Sigma-Aldrich) were applied to treat the HEI-OC1 cells individually. The protein of the apoptosis-related gene was detected by western blot, and the expression level was determined to identify the target neomycin concentration to be used in the following experiments. D-JNKI-1 (2 μM) was applied to HEI-OC1 cells. Four groups were included in this study: HEI-OC1, HEI-OC1 + D-JNKI-1, HEI-OC1 + neo, and HEI-OC1 + neo + D-JNKI-1. HEI-OC1 was a normal control (NC) group treated with an equivalent volume of DMSO as a substitute for D-JNKI-1 and neomycin. Neomycin and D-JNKI-1 were applied through the entire process and incubated under identical conditions. The following markers were measured: DCFH-DA assay for cellular ROS level, CCK8 for cell viability; western blot and immunofluorescence staining for the protein expression of apoptosis markers, such as Bcl-2, Bax, caspase-3, and caspase-8. HEI-OC1 cell apoptosis was detected by terminal deoxynucleotidyl transferase (TdT)-mediated dUTP-biotin nick end labeling (TUNEL) staining and flow cytometry.

### House Ear Institute-Organ of Corti 1 Cell Line Culture

House ear institute-organ of corti 1 cells were cultured in Dulbecco’s modified Eagle’s medium (DMEM) containing 10% fetal bovine serum (FBS) and incubated under permissive conditions (33°C and 5% CO2) (Kalinec et al., 2003). Experiments were performed when cell growth reached approximately 80% confluence. The target concentration of neomycin with or without D-JNKI-1 was applied to the cells.

### Western Blot to Identify Protein Expression

House ear institute-organ of corti 1 cells from control and treated as above the procedure described were collected, and western blotting was performed. Briefly, total proteins from treated HEI-OC1 cells were extracted using fresh buffer made up with western and IP lyse and PMSF on ice for 10 min, centrifuged, and supernatants collected to quantify the protein concentration. Equal amounts of proteins (20 μg) were loaded on, separated by 8–15% SDS-PAGE electrophoresis, and transferred to polyvinylidene fluoride membranes (PVDFs). The membranes were blocked with 5% non-fat dried milk in TBS-T with 0.1% Tween 20 for 60 min at room temperature and were incubated overnight at 4°C with primary antibodies, including mouse anti-Bcl-2 (sc-7382, Santa Cruz, United States), rabbit anti-Bax (50599-2-ig, Proteintech), rabbit anti-cleaved caspase-3 (19677-1-AP, Proteintech), rabbit anticaspase-8 (13423-1-AP, Proteintech), rabbit anti-AMPKɑ(5832S, CST, United States), rabbit anti-p-AMPKɑ(2535S, CST, United States), rabbit anti-JNK1 (ab179461, Abcam, United States), rabbit anti-P38 (8690S, CST, United States), rabbit anti-p-P38 (bs-5476R, Bioss, China), and mouse anti-GAPDH (60004-1-ig, Proteintech). The membranes were incubated with a secondary IgG antibody conjugated to horseradish peroxidase (HRP) (1:3,000) for 1 h. Immunoreactivity was detected with an enhanced chemiluminescence (ECL) detection system. The gray level of protein bands was quantified using ImageJ software (Broken Symmetry Software, United States). GAPDH was used as a standard protein expression for normalization.

### Evaluation of the Concentration of Neomycin-Induced House Ear Institute-Organ of Corti 1 Cell Apoptosis

The target concentration of neomycin was determined by the protein expression level of HEI-OC1 cell apoptosis induced by neomycin. After initial incubation, HEI-OC1 cells were treated with different concentrations of neomycin at 0.5 mM, 1.0 mM, 2.0 mM, and 5.0 mM for 24 h individually. The apoptosis markers, such as Bax, Bcl-2, caspase-3, and caspase-8, in the neomycin-treated HEI-OC1 cells were detected by western blot. The expression level of the protein was detected and measured. The target neomycin concentration was determined according to the neomycin-induced apoptotic protein expression level. Once the neomycin concentration was determined, it will be used in the following experiments.

### Cell Viability Measurement

Cells with or without exposure to the target concentration of neomycin and D-JNKI-1 were seeded in 96-well plates. Cell viability was measured using the Cell Counting CCK-8 Kit (CCK-8, Meilunbio, China) according to the manufacturer’s instructions. About 10 μl of CCK-8 solution was added to each well and incubated at 37°C for 30 min. The optical density (OD) at the absorbance 450 nm was recorded by a microplate reader (Bio-Rad).

### Immunofluorescence Staining

Treated cells were fixed with 4% paraformaldehyde for 30 min and then incubated in a blocking medium (goat serum, 1% Triton X-100) at room temperature. After 30 min, cells were incubated with primary antibodies, such as Bax, Bcl-2, caspase-3, and caspase-8, for 2 h at 4°C. After washing three times with PBST, the cells were incubated with the secondary antibody for 1 h at room temperature. Cell nuclei were counterstained with DAPI for 10 min. The samples were washed and mounted on slides. The samples were imaged with an LSM700 confocal microscope.

### Flow Cytometry

Annexin V-FITC was used to segregate and quantitate the apoptotic cells using flow cytometry. HEI-OC1 cells were incubated with the Annexin V-FITC Kit (eBioscience, 88-8007-74) according to the instructions. Briefly, treated HEI-OC1 cells were collected, washed two times with cold PBS, and then resuspended in 1 × binding buffer at a concentration of 1 × 10^6^ cells/ml. About 5 μl annexin V-FITC and 5 μl PI were added and gently mixed with 100 μl cell suspension (1 × 10^5^ cells) and incubated for 15 min at room temperature in the dark. A total volume of 400 μl 1 × binding buffer was added to the tubes. The samples were analyzed by fluorescence-activated cell sorting (Becton, United States) as soon as possible. Double-positive propidium iodide and annexin-V staining were assigned as late-stage apoptotic cells, and double-negative staining was assigned as a viable cell. The early apoptotic cells were stained with annexin-V staining, and necrotic cells were stained with PI only. Total apoptosis includes early- and late-stage apoptosis. All tests were repeated four times independently.

### Terminal Deoxynucleotidyl Transferase-Mediated dUTP-Biotin Nick End Labeling

The TUNEL staining was conducted utilizing the Click-iT^®^ Plus TUNEL Assay (Life Technologies, United States) according to the manufacturer’s protocol. Briefly, HEI-OC1 cells were fixed with 4% PFA in PBS for 30 min, permeabilized with 0.1% Triton X-100 in PBS for 10 min, and stained with TUNEL working solution for 1 h at 37°C in the dark. Cell nuclei were counterstained with DAPI. Stained cells were visualized, and apoptotic cells were determined by using a confocal laser scanning microscopy.

### Reactive Oxygen Species Detection

The intracellular ROS level was measured by DCFH-DA staining (D6883, Sigma Technologies) according to the manufacturer’s instructions. Briefly, cells treated with the designated conditions were incubated with 10 μM DCFH-DA in a serum-free medium for 30 min. Fluorescent signal intensity was detected under fluorescence microscopy.

### Statistical Analysis

Data were statistically analyzed using GraphPad Prism 9.0. One-way and two-way ANOVAs followed by Tukey’s multiple comparisons *post hoc* test were used when comparing more than two groups. The unpaired *t*-test was when comparing two groups. All values are shown as the mean ± standard deviation (SD), and *p* < 0.05 was considered a statistically significant difference.

## Results

### Neomycin Concentration Dose-Dependently Induced Apoptotic Protein Expression in House Ear Institute-Organ of Corti 1 Cells

**FIGURE 1:**
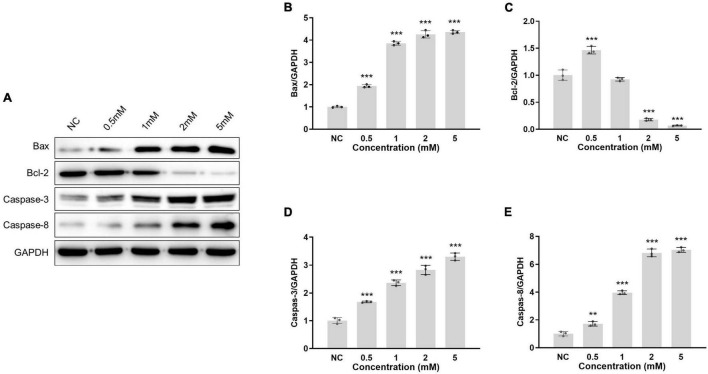
Protein of apoptotic-related gene expression in HEI-OC1 cells treated with different neomycin concentrations. **(A)**: HEI-OC1 cells were treated with 0.5, 1.0, 2.0, and 5.0 mM neomycin, the protein expressions of Bax, Bcl-2, caspase-3, and caspase-8 in cells were detected by western blot. The expression level was the gray value of the protein band normalized to GAPDH. The protein expression levels of Bax **(B)**, Bcl-2 **(C)**, caspase-3 **(D)**, and caspase-8 **(E)** were measured at different neomycin concentrations. One-way ANOVA, **p < 0.01, ***p < 0.001, comparison the expression of protein in different concentrations of neomycin treatment and normal (NC). Data are presented as mean ± SD. All tests were repeated three times independently.

Neomycin-induced HEI-OC1 cell apoptosis was used to determine the experimental neomycin concentration. Four different concentrations of neomycin were selected for 0.5 mM, 1.0 mM, 2.0 mM, and 5.0 mM. Apoptotic-related proteins were detected by western blot (Figure 1A). The protein expression level of neomycin-induced apoptosis was evaluated and selected as experiment target concentration. Apoptotic protein expression levels of Bax, caspase-3, and caspase-8 were increased, and antiapoptotic protein Bcl-2 expression level was decreased following neomycin concentration increase (Figures 1B–E). Compared to normal control, the expression levels showed dose-dependent effect and significant change among all four proteins treated at 2.0 mM neomycin exposure, and the expression level presented similarly above 2mM; thus, 2.0 mM neomycin was appropriate and selected as the target concentration since it induced the strongest apoptotic reaction and did not damage cells too much. This concentration was used for subsequent experiments.

### D-JNKI-1 Suppressed Neomycin-Induced Reactive Oxygen Species Generation in House Ear Institute-Organ of Corti 1 Cells

**FIGURE 2:**
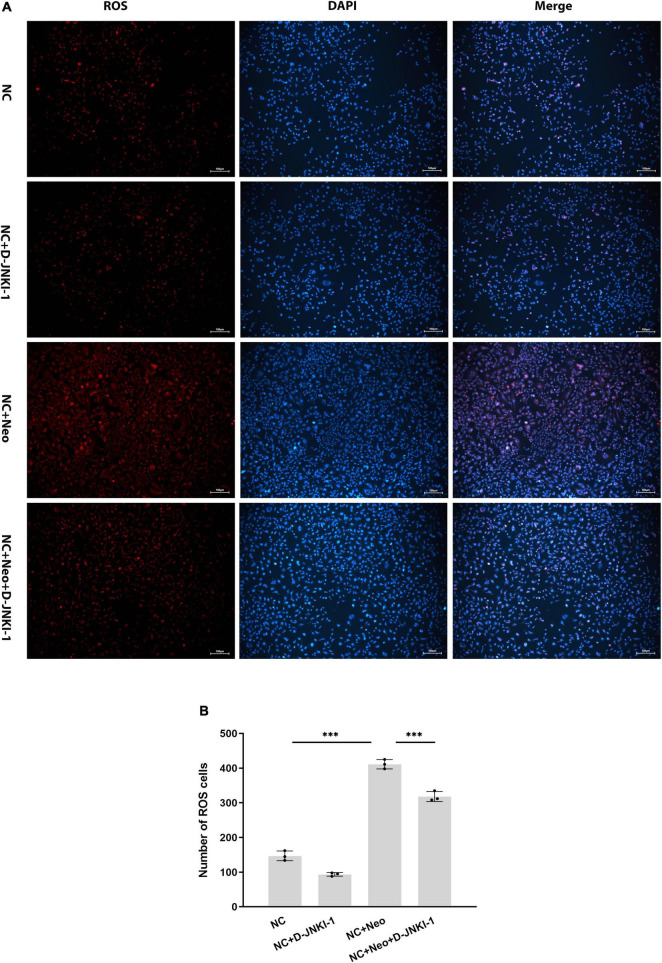
Reactive oxygen species immunostaining exhibition on neomycin- and D-JNKI-1-treated HEI-OC1 cells. DCFH-DA staining was used to detect ROS in HEI-OC1 cells. **(A)**: The ROS-positive staining is red, scale bar = 100 μm. The ROS staining cells with D-JNKI-1 (2 μM) treatment show fewer than that of the normal control group. ROS in HEI-OC1 cells exposed to neomycin was increased, and D-JNKI-1 treatment reduced the HEI-OC1 cell ROS production. **(B)** Comparison of the numbers of ROS-positive staining cells, one-way ANOVA, ^***^p < 0.001, with data presented as mean ± SD. All tests were repeated three times independently.

To examine the D-JNKI-1 effect on neomycin-induced ROS, D-JNKI-1 was applied to neomycin-treated HEI-OC1 cells. Figure 2A shows fewer ROS-positive staining exhibited in the non-treated and D-JNKI-1-treated HEI-OC1 cells, suggesting that D-JNKI-1 did not increase intracellular ROS formation. In contrast to the normal control, intracellular ROS were significantly increased with neomycin treatment (Figure 2B); however, D-JNKI-1 coadministration with neomycin significantly suppressed neomycin-induced ROS formation. Taken together, D-JNKI-1 presented a protective effect against neomycin-induced ROS production.

### D-JNKI-1 Ameliorated Neomycin Decreased House Ear Institute-Organ of Corti 1 Cell Viability

**FIGURE 3:**
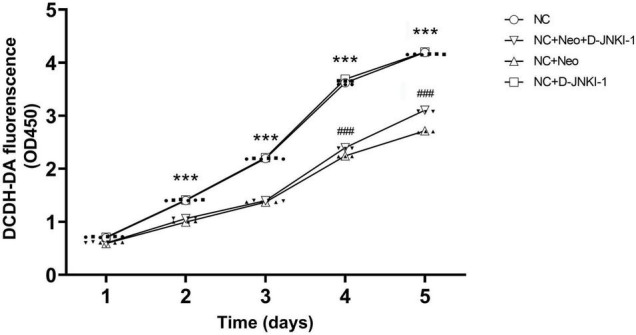
Time-dependent manner of HEI-OC1 cell viability treated with D-JNKI-1 and neomycin. Four groups of HEI-OC1 cells were treated with D-JNKI-1 (2 μM) and neomycin. Cell viability was examined using a CCK-8 cell counting kit and was measured on days 1, 2, 3, 4, and 5. Detection absorbance at 450 nm was determined at the test time points and 650 nm was used as the reference. Two-way ANOVA (treatment × time factors), ***p < 0.001, comparing NC + Neo group to NC group, ^###^*p* < 0.001, comparing NC + Neo + JNKI-1 group to NC + Neo group, with data presented as mean ± SD. All tests were repeated three times independently.

To examine the effect of D-JNKI-1 on HEI-OC1 cell viability, we measured the cell viability at different time points using the CCK-8 assay. Cell viabilities were measured at five time points, and Figure 3 shows, in HEI-OC1 cells treated with D-JNKI-1, that the cell viability was similar to the HEI-OC1 cell-only control group, indicating that D-JNKI-1 did not result in HEI-OC1 cell ototoxicity. In HEI-OC1 cells treated with 2 mM neomycin, the cell viability decreased significantly, indicating neomycin-induced HEI-OC1 cell cytotoxicity. The cell viability was gradually increased as D-JNKI-1 was applied to the neomycin-treated HEI-OC1 cells, and the cell viability was significantly increased after 4 days. The results showed that D-JNKI-1 significantly increased cell viability and ameliorated neomycin-induced HEI-OC1 cell death.

### D-JNKI-1 Protects House Ear Institute-Organ of Corti 1 Cells Against Neomycin-Induced Cell Damage

**FIGURE 4:**
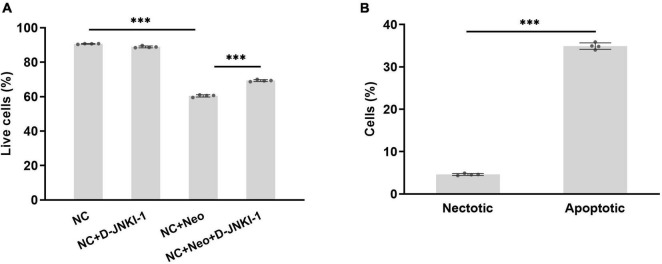
Percentage of surviving HEI-OC1 cells treated with D-JNKI-1 and neomycin. Live and damaged cells were measured with flow cytometry. Double-positive propidium iodide and annexin-V staining were assigned as late-stage apoptotic cells, and double-negative staining was assigned as viable cells. The early apoptotic cells were stained with annexin-V staining, and necrotic cells were stained with PI only. Total apoptosis includes early- and late-stage apoptosis. **(A)** percentage of alive cells in various groups, one-way ANOVA, ^***^p < 0.001; **(B)** compared percentage of necrotic and apoptotic cells with neomycin treatment, two-tailed, unpaired *t*-test, ^***^p < 0.001, data are presented as mean ± SD. All tests were repeated four times independently.

House ear institute-organ of corti 1 cell viability was suppressed with neomycin treatment. To know the effect of D-JNKI-1 on neomycin-damaged HEI-OC1 cells, the percentage of death and survival cells treated or untreated were detected by flow cytometry and calculated. The results show that the percentage of cell death and survival were not affected by D-JNKI-1 treatment compared to the normal control group, and the percentage of survival HEI-OC1 cells was significantly decreased after neomycin exposure and increased with neomycin and D-JNKI-1 treatment simultaneously (Figure 4A). The damaged HEI-OC1 cells caused by neomycin were mainly apoptotic cells (Figure 4B). The results showed that neomycin significantly induced HEI-OC1 cell death and indicated that D-JNKI-1 had protective effects against neomycin-induced HEI-OC1 cell damage.

### D-JNKI-1 Reduced Neomycin-Induced House Ear Institute-Organ of Corti 1 Cell Apoptosis

**FIGURE 5:**
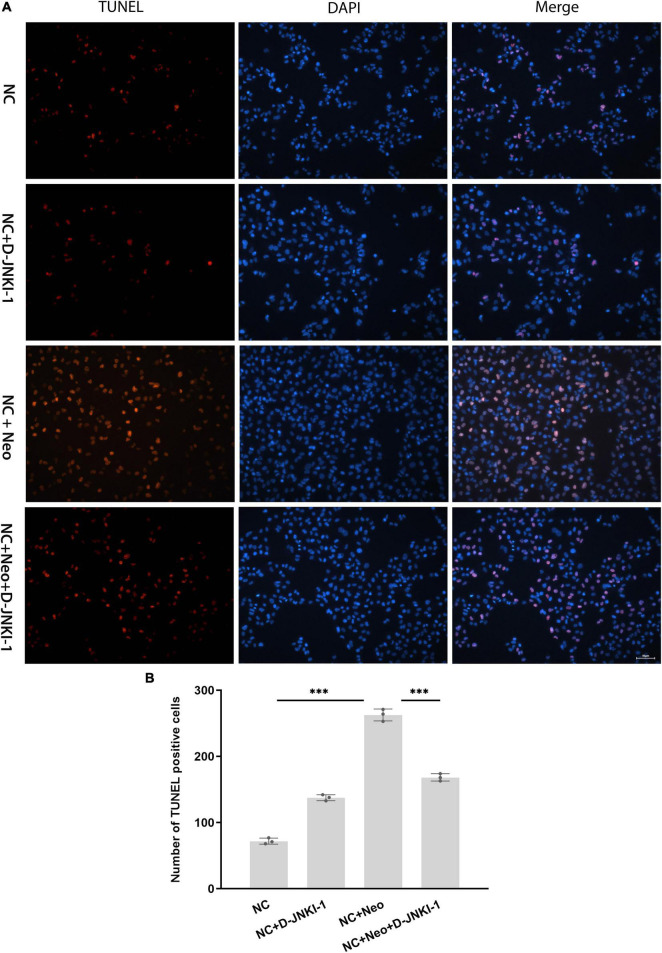
Apoptosis expression in D-JNKI-1 and neomycin-treated HEI-OC1 cells. Apoptotic cells were detected using TUNEL staining. **(A)** The TUNEL-positive staining is red and presented in different groups, scale bar = 50 μm. **(B)** The number of TUNEL-positive cells was significantly increased by exposure to neomycin, and TUNEL-positive cell formation was significantly decreased with D-JNKI-1 treatment. One-way ANOVA, ^***^p < 0.001, data are presented as mean ± SD. All tests were repeated three times independently.

To determine whether D-JNKI-1 could prevent neomycin-induced HEI-OC1 cell apoptosis, the TUNEL assay and DAPI staining were used to examine neomycin-induced HEI-OC1 cell apoptosis. Figures 5A,B show, compared to the control group, TUNEL-positive cells were significantly increased with neomycin exposure, and D-JNKI-1 and neomycin co-treatment significantly decreased TUNEL-positive cells. The results confirmed that D-JNKI-1-inhibited neomycin promoted apoptotic signaling formation and reduced neomycin-induced HEI-OC1 cell apoptosis.

**FIGURE 6:**
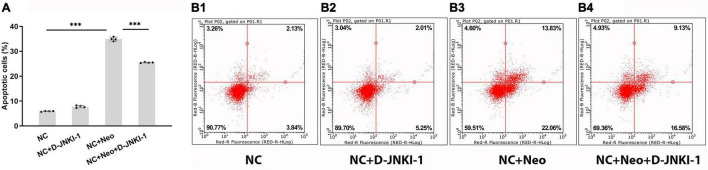
Percentage of apoptosis change with D-JNKI-1 effect on neomycin-exposed HEI-OC1 cells. Apoptosis was assigned with annexin V-FITC and PI double staining. Quantification of percentage of apoptosis was shown **(A)**. Late apoptotic cells (double-positive propidium iodide and annexin-V staining) were shown in the upper right quadrant (UR), and early apoptotic cells (annexin-V staining) were plotted in the lower right quadrant (LR). Histograms of four groups were exhibited **(B1–4)**. One-way ANOVA, ^***^p < 0.001, data are represented as mean ± SD. All tests were repeated four times independently.

House ear institute-organ of corti 1 cells were treated with neomycin with or without co-treatment of D-JNKI-1, and apoptotic cells were identified and quantified using flow cytometry. The percentage of apoptotic cells were analyzed using annexin V-FITC and PI double staining. The total number of apoptotic cells counted as late apoptotic cells (UR) and early apoptotic cells (LR) is shown in the histograms (Figures 6B1–4). The number of apoptotic cells co-treated neomycin with D-JNKI-1 (mean 25.5%) was significantly decreased compared to neomycin-only (mean 34.9%, *p* < 0.001) (Figure 6A). The results showed that D-JNKI-1 coadministration significantly inhibited neomycin-promoting HEI-OC1 cell apoptosis (*p* < 0.001).

### D-JNKI-1 Suppressed Neomycin-Induced House Ear Institute-Organ of Corti 1 Cell Apoptotic Protein Expression

**FIGURE 7:**
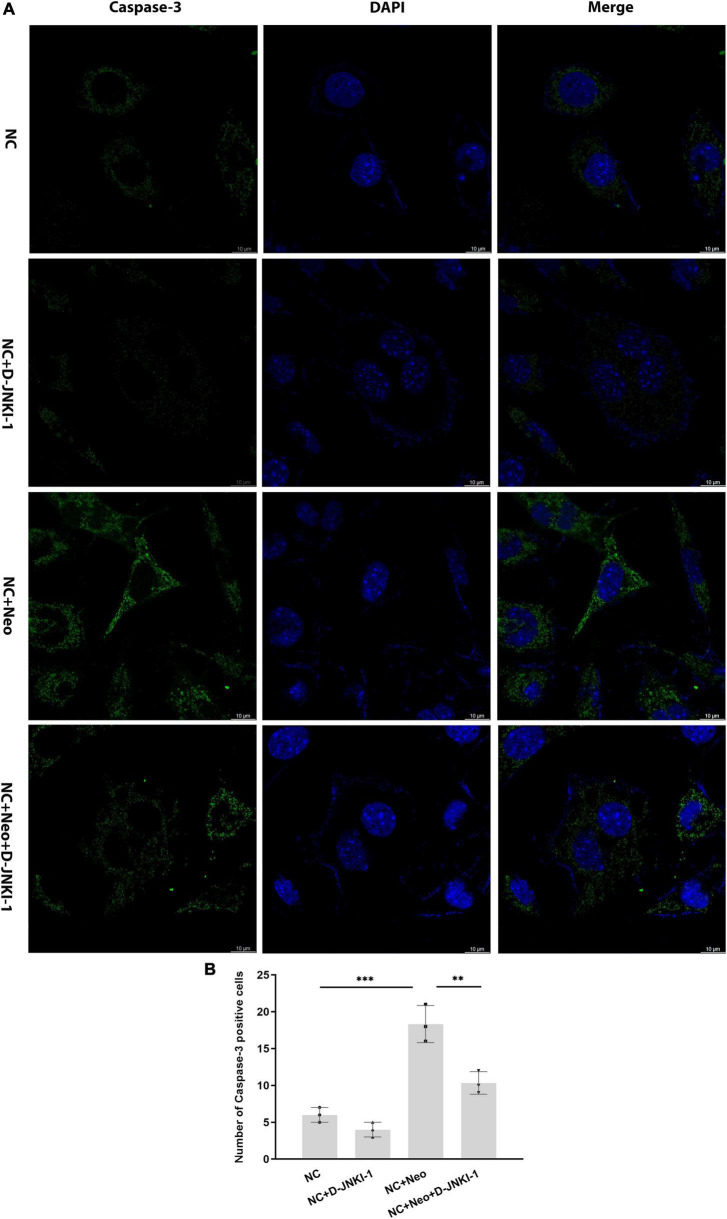
Caspase-3 expression on D-JNKI-1 and neomycin-treated HEI-OC1 cells. Caspase-3 was detected by immunostaining (green). **(A)** The caspase-3-positive staining cells were observed after neomycin exposure. D-JNKI-1 treatment reduced the caspase-3-positive staining, scale bar = 10 μm. **(B)** The number of caspase-3-positive cells exhibited in the HEI-OC1 cells treated with neomycin and D-JNKI-1, one-way ANOVA, ^**^p < 0.01, ^***^p < 0.001, data are presented as mean ± SD. All tests were repeated three times independently.

**FIGURE 8:**
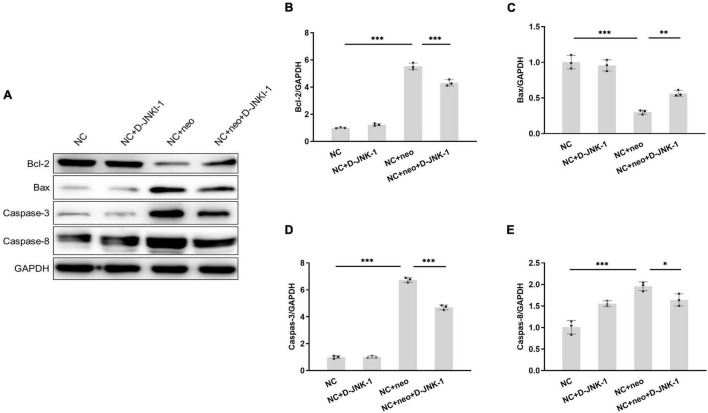
Protein expression of apoptosis-related genes exhibits on D-JNKI-1 and neomycin-treated HEI-OC1 cells. Protein expressions of apoptosis-related genes were detected by western blot **(A)**. Bax, Bcl-2, caspase-3, and caspase-8 proteins were detected. The protein expression level was measured with each band gray scale value, normalized to GAPDH levels **(B–E)**. One-way ANOVA, *p < 0.05, ^**^p < 0.01, ^***^p < 0.001, data are presented as mean ± SD. All tests were repeated three times independently.

The apoptotic-related proteins were detected to assess the effect of D-JNKI-1 on the neomycin-induced HEI-OC1 cell apoptosis. The caspase-3 in HEI-OC1 cells was detected by immunostaining. As shown in Figures 7A,B, positive caspase-3 immunostaining was detected in cells with neomycin treatment and reduced with D-JNKI-1 treatment. The protein expression levels of Bax, Bcl-2, caspase-3, and caspase-8 were detected by western blot in Figure 8A. Figures 8B–E show that the expression level of Bcl-2 was decreased with neomycin treatment, and D-JNKI-1 inhibited the decreased expression level. The protein expression levels of Bax, caspase-3, and caspase-8 were increased with neomycin treatment, and D-JNKI-1 treatment suppressed the expression of Bax and caspase-3. Taken together, the protein expression illustrated the D-JNKI-1 effect on the neomycin-induced apoptotic response and indicated that D-JNKI-1 acted as a JNK inhibitor to reduce neomycin-induced HEI-OC1 cell apoptosis.

### D-JNKI-1 Reduced Neomycin-Induced House Ear Institute-Organ of Corti 1 Cell Apoptosis by JNK Activation and AMPKɑand p38 Phosphorylation

**FIGURE 9:**
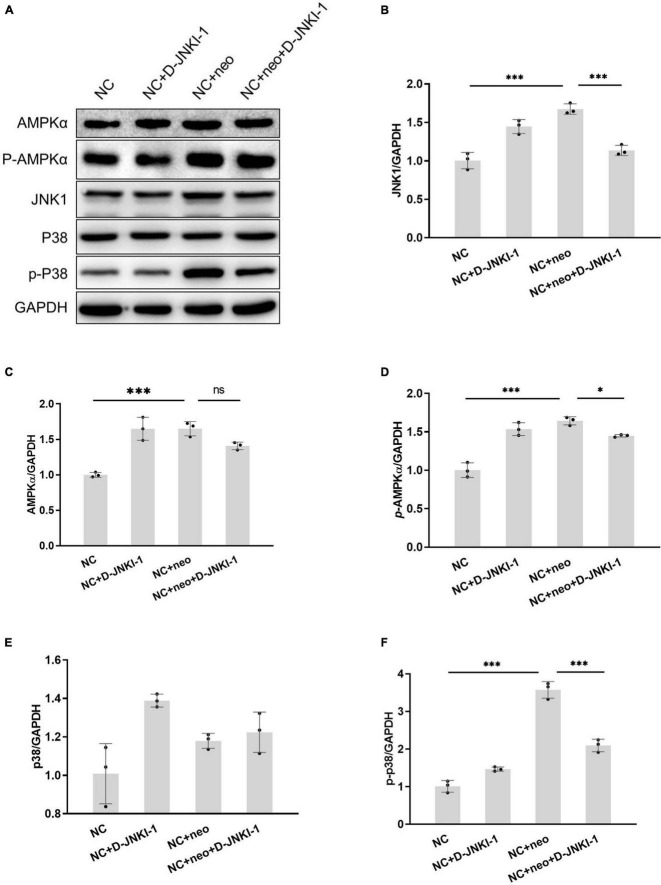
D-JNKI-1 inhibition involved in neomycin-induced HEI-OC1 cell apoptosis pathway. The JNK pathway related to apoptosis was detected. The protein expressions of JNK1, AMPKɑ, p-AMPKɑ, p38, and p-P38 were detected by western blot **(A)**. The protein expression level was measured according to each band gray scale value, normalized to GAPDH level **(B–F)**. One-way ANOVA, *p < 0.05, ^***^p < 0.001, data are presented as mean ± SD. All tests were repeated three times independently.

Since D-JNKI-1 has been implicated in apoptosis inhibition, we detected signaling pathway proteins related to apoptosis to investigate the effects of D-JNKI-1 on neomycin-induced HEI-OC1 cell apoptosis. JNK activation in neomycin-treated cells was detected by western blot. Figure 9 shows that the protein expression levels of JNK1, p-AMPKɑ, and p-p38 were significantly increased after neomycin treatment, and the expression levels were decreased after D-JNKI-1 was applied simultaneously. The protein expression levels of AMPKa and p38 were not different between the NC + Neo and the NC + Neo + JNKI-1 groups. The results suggested that D-JNKI-1 inhibits apoptosis by inhibiting JNK1 activation and AMPKɑand p38 phosphorylation to protect against neomycin-induced cell apoptosis.

## Discussion

Hair cells are susceptible to damage from aminoglycosides. Aminoglycosides induce hair cell oxidative stress; ROS levels in hair cells exposed to neomycin revealed the aminoglycoside-induced ototoxicity in hair cells and ROS accumulation in hair cells leads to cell death. Aminoglycoside as an ototoxic drug-induced apoptotic character in hair cell death (Matsui et al., 2002). We used hair cell-like HEI-OC1 cells in this study. An extremely high concentration (2 mM) of neomycin was applied for 24 h to induce apoptosis because HEI-OC1 cells lack the mechano-electrical transduction channels that form the entry route for aminoglycosides into hair cells (Kenyon et al., 2017). Our results were consistent with reports showing that ROS production in HEI-OC1 cells increased after exposure to neomycin.

The TUNEL staining and western blotting revealed that HEI-OC1 cells exposed to neomycin exhibit characteristics of apoptosis. Apoptosis is a highly regulated process of cell death, including extrinsic and intrinsic apoptosis. The extrinsic apoptotic cell death program executed by caspase-8 initiated a cascade to active caspase-3 by cleavage of proteins, ultimately leading to cellular degeneration. Our studies support that HEI-OC1 cell apoptosis involved in neomycin activates caspase-8 and cleaves caspase-3 (Jiang et al., 2006).

Multiple mechanisms are involved in apoptosis induction (Wang and Puel, 2018). The previous study has been reported that aminoglycoside-induced hair cell apoptosis is related to the JNK signaling pathway (Wang et al., 2003). The intrinsic pathway is the major apoptotic pathway initiated by aminoglycoside ototoxicity. In addition to being involved in mitochondria mediation, the JNK signaling pathway can be activated by accumulated high levels of toxic ROS and contributes to hair cell apoptosis (Gupta et al., 1995; Davis, 2000). JNK activation and phosphorylation are important for the activation of proapoptotic family Bcl-2 proteins (Harris and Johnson, 2001; Whitfield et al., 2001). Adenosine 5′-monophosphate-activated protein kinase (AMPK) is a regulator of cellular and organismal metabolism (Carling and Viollet, 2015). ROS triggered AMPK hyperactivation in response to cellular energy results in mitochondrial dysfunction. ROS accumulation and apoptosis formation promoting signaling increase were associated with AMPK activation in cochlear tissues (Zhao et al., 2020). AMPK is also related to JNK; through a positive feedback mechanism, AMPK suppression inhibits the JNK proapoptotic cascade associated with ROS (Yun et al., 2009). This study was consistent with reports showing that neomycin exposure induced the ROS overgeneration, downregulated Bcl2, upregulated Bax, and activated JNK. Additionally, our results found out that neomycin-induced AMPKα phosphorylation increased in HEI-OC1 cells, which indicated that HEI-OC1 cell stress was caused by ROS generation, and through this intracellular stress-mediated apoptosis pathways lead to HEI-OC1 cell apoptosis.

D-JNKI-1 served as a JNK inhibitor to interfere with neomycin-induced apoptosis. As JNK phosphorylation can be triggered by ototoxic stress (Anttonen et al., 2016), treatment of neomycin-exposed explants with D-JNKI-1 blocked the MAPK–JNK-evoked increase in *c-fos* mRNA (Wang et al., 2003). Our study showed that D-JNKI-1 decreased neomycin-induced HEI-OC1 cell ROS generation, it inhibited JNK and p38 to suppress caspase-8 and caspase-3, and D-JNKI-1 affected mitochondrial antiapoptotic gene Bcl-2 and proapoptotic gene Bax activation to reduce HEI-OC1 cell apoptosis. Our results support that D-JNKI-1 suppressed JNK phosphorylation to attenuate cellular injury in the Cochlea and blocked JNK-mediated phosphorylation of c-Jun to inhibit ototoxic hair cell death (Bonny et al., 2001). The previous study has been reported that AMPK is related to noise-induced hair cell damage (Hill et al., 2016). Our study found out that D-JNKI-1 decreased neomycin-induced AMPK phosphorylation in HEI-OC1 cells. These results revealed that D-JNKI-1 protects against neomycin-induced apoptosis by suppressing ROS overproduction triggered JNK activation and p38 and AMPK phosphorylation, maintaining mitochondrial function and inhibiting cell damage. It suggested that both extrinsic and intrinsic pathways were involved in D-JNKI-1 inhibiting neomycin-induced HEI-OC1 cell apoptosis.

In conclusion, the data demonstrate that neomycin contributes to ROS-induced apoptosis in HEI-OC1 cells. D-JNKI-1 protects against HEI-OC1 damage from neomycin by suppressing ROS generation, inhibiting JNK activation and p38 and AMPK phosphorylation. These results suggest that D-JNKI-1 application attenuated neomycin-induced HEI-OC1 oxidative stress and apoptosis and may provide a potential therapy to reduce aminoglycoside-induced ototoxicity. D-JNKI-1 local delivery blocking MAPK-JNK pathway to rescue hair cells and restore hearing function from noise and aminoglycoside exposure *in vivo* has been reported (Wang et al., 2003), D-JNKI-1 systemic delivery blocking JNK and AMPK signaling pathway to reduce aminoglycoside ototoxicity can be further investigated.

## Data Availability Statement

The original contributions presented in the study are included in the article/supplementary material, further inquiries can be directed to the corresponding author/s.

## Author Contributions

JZ designed the experiments and wrote the article. JZ, ZH, HL, and RY conducted the experiments. JZ, ZH, HL, RY, and LG analyzed the data. JZ, HL, and LG reviewed and edited the article. All authors contributed to the article and approved the submitted version.

## Conflict of Interest

The authors declare that the research was conducted in the absence of any commercial or financial relationships that could be construed as a potential conflict of interest.

## Publisher’s Note

All claims expressed in this article are solely those of the authors and do not necessarily represent those of their affiliated organizations, or those of the publisher, the editors and the reviewers. Any product that may be evaluated in this article, or claim that may be made by its manufacturer, is not guaranteed or endorsed by the publisher.
